# Differential responses of *Lasiopodomys mandarinus* and *Lasiopodomys brandtii* to chronic hypoxia: a cross-species brain transcriptome analysis

**DOI:** 10.1186/s12864-018-5318-1

**Published:** 2018-12-11

**Authors:** Qianqian Dong, Luye Shi, Yangwei Li, Mengwan Jiang, Hong Sun, Baishi Wang, Han Cheng, Yifeng Zhang, Tian Shao, Yuhua Shi, Zhenlong Wang

**Affiliations:** 1 School of Life Sciences, Zhengzhou University, Zhengzhou, 450001 Henan China; 2 Ministry of Education Key Laboratory for Biodiversity Science and Ecological Engineering College of Life Sciences, Beijing Normal University, Beijing, 100875 China; 3 Central Laboratory, The Affiliated Cancer Hospital of Zhengzhou University, Zhengzhou, 450008 Henan China; 4 Institute of Forensic Science, Ministry of Public Security, Beijing, 100038 China

**Keywords:** *Lasiopodomys mandarinus*, *Lasiopodomys brandtii*, Chronic hypoxia, Transcriptome analysis, Brain

## Abstract

**Background:**

Subterranean rodents have evolved many features to adapt to their hypoxic environment. The brain is an organ that is particularly vulnerable to damage caused by exposure to hypoxic conditions. To investigate the mechanisms of adaption to a hypoxic underground environment, we carried out a cross-species brain transcriptome analysis by RNA sequencing and identified genes that are differentially expressed between the subterranean vole *Lasiopodomys mandarinus* and the closely related above-ground species *Lasiopodomys brandtii* under chronic hypoxia [10.0% oxygen (O_2_)] and normoxia (20.9% O_2_).

**Results:**

A total of 355 million clean reads were obtained, including 69,611 unigenes in *L. mandarinus* and 69,360 in *L. brandtii*. A total of 235 and 92 differentially expressed genes (DEGs) were identified by comparing the hypoxic and control groups of *L. mandarinus* and *L. brandtii*, respectively. A Gene Ontology (GO) analysis showed that upregulated DEGs in both species had similar functions in response to hypoxia, whereas downregulated DEGs in *L. mandarinus* were enriched GO terms related to enzymes involved in aerobic reactions. In the Kyoto Encyclopedia of Genes and Genomes pathway analysis, upregulated DEGs in *L. mandarinus* were associated with angiogenesis and the increased O_2_ transport capacity of red blood cells, whereas downregulated DEGs were associated with immune responses. On the other hand, upregulated DEGs in *L. brandtii* were associated with cell survival, vascular endothelial cell proliferation, and neuroprotection, while downregulated genes were related to the synaptic transmission by neurons.

**Conclusions:**

*L. mandarinus* actively adapts its physiological functions to hypoxic conditions, for instance by increasing O_2_ transport capacity and modulating O_2_ consumption. In contrast, *L. brandtii* reacts passively to hypoxia by decreasing overall activity in order to reduce O_2_ consumption. These results provide insight into hypoxia adaptation mechanisms in subterranean rodents that may be applicable to humans living at high altitudes or operating in other O_2_-poor environments.

**Electronic supplementary material:**

The online version of this article (10.1186/s12864-018-5318-1) contains supplementary material, which is available to authorized users.

## Background

Oxygen (O_2_) is essential to sustain most aerobic organisms, and O_2_ deprivation creates a significant physiological stress. Hypoxia occurs under natural conditions, for instance in underground tunnels [1], at high altitude [2], in aquatic habitats [3], and in the tumor microenvironment [4, 5]. For many vertebrates, disruption of the O_2_ supply to the brain for more than a few minutes leads to irreversible neurological damage due to neuronal death [6]. Species that inhabit subterranean environments have developed effective strategies to survive under hypoxia and exhibit a variety of convergent morphological, physiological, behavioral, and genomic adaptations [7–9].

Recent studies have revealed the molecular mechanisms underlying the response to hypoxia in subterranean mammals, including the differential expression of genes encoding hemoglobin, hypoxia inducible factor (HIF)-1α, erythropoietin, and vascular endothelial growth factor (VEGF) [10–12]. The expression patterns of these genes differ between subterranean mammals and mammals living above ground; the former have evolved a unique cardiovascular system as an adaptation of hypoxia. A large-scale transcriptome sequencing study of the blind mole rat (*Spalax galili*), a typical subterranean rodent, revealed that apoptosis was suppressed and the expression of angiogenic factors was tightly regulated in the hypoxic environment [13, 14]. However, it is possible that different species of subterranean rodents have evolved distinct adaptive mechanisms in response to chronic hypoxia [15, 16].

The Mandarin vole (*Lasiopodomys mandarinus*) is a subterranean rodent that is widely distributed throughout northeast and central China and north central Mongolia, as well as in the adjacent areas of Siberia south of Lake Baikal and the southern and central Korean Peninsula. For most of its life, *L. mandarinus* lives in an underground tunnel system characterized by chronic hypoxia and darkness. As a subterranean species, *L. mandarinus* exhibits remarkable physiological adaptations to hypoxic stress including a higher capillary density and elevated values of blood parameters such as hematocrit, mean corpuscular volume, mean corpuscular hemoglobin (MCH), and MHC concentration (MCHC) [17]. As the sibling species of *L. mandarinus*, Brandt’s vole (*L. brandtii*) is mainly distributed in the grasslands of middle-eastern Inner Mongolia, eastern regions of Mongolia, and some parts of southern Russia [18]. Unlike *L. mandarinus*, *L. brandtii* spends most of its life above ground. Their close evolutionary relationship and distinct life histories make *L. mandarinus* and *L. brandtii* ideal animal models for a comparative study of mechanisms of adaption to hypoxia in subterranean mammals.

In the present study, we sequenced and assembled the brain transcriptomes of *L. mandarinus* and *L. brandtii* under conditions of chronic hypoxia and normoxia. Whole brain RNA was extracted and subjected to Illumina sequencing to identify genes that are differentially expressed under the two conditions as well as between the two species.

## Results

### Illumina sequencing and de novo transcriptome assembly

A total of 355 million reads with 89.4 billion bases were obtained after stringent quality assessment and data filtering (Additional file 1: Table S2). We obtained 144,789 transcripts (mean length: 1989.39) and 69,611 unigenes (mean length: 944.46) with an N50 of 2214 for *L. mandarinus*, and 167,002 transcripts (mean length: 2501.43) and 69,360 unigenes (mean length: 974.89) with an N50 of 2306 for *L. brandtii*. The length distribution of assembled unigenes is shown in Additional file 1: Table S3.

### Functional annotation

**Fig. 1:**
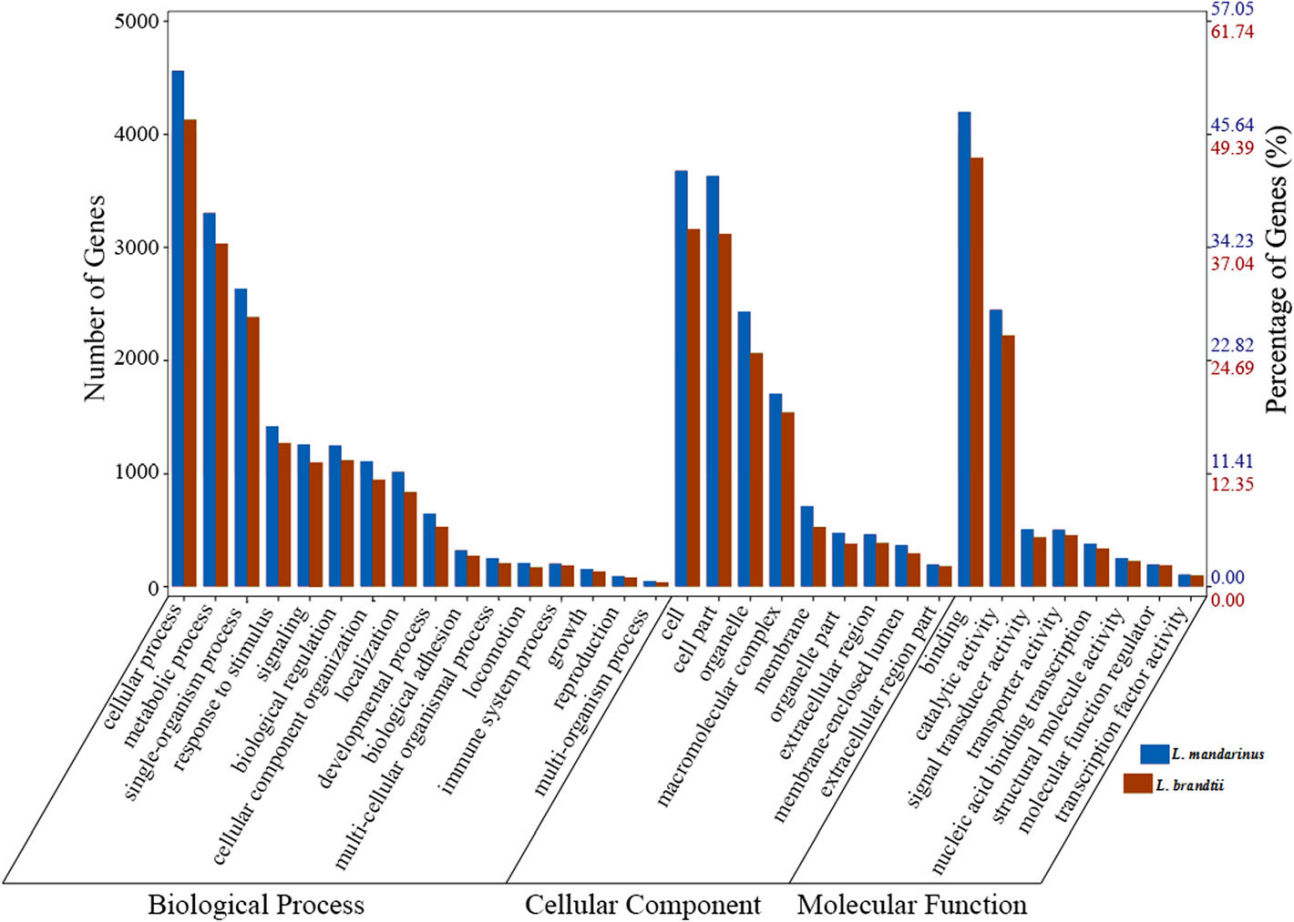
GO annotations for *L. mandarinus* and *L. brandtii* transcriptomes

According to the BLASTX results, 20,011 (28.75%) unigenes of *L. mandarinus* and 19,120 (27.70%) unigenes of *L. brandtii* had homologous proteins in the NCBI non-redundant (Nr) database (Additional file 1: Table S4). Based on annotated unigenes in the database, 10,593 and 9838 unigenes of *L. mandarinus* and *L. brandtii*, respectively, were assigned to one or more Gene Ontology (GO) terms, with 33.5%/32.5% in cellular components, 21.1%/21.7% in molecular functions, and 45.4%/45.8% in biological processes (Fig. 1). To identify biological pathways that are differentially regulated between *L. mandarinus* and *L. brandtii*, the annotated unigenes were mapped to reference pathways in the Kyoto Encyclopedia of Genes and Genomes (KEGG) database. *L. mandarinus* and *L. brandtii* unigenes were mapped to 368 pathways.

### Gene expression pattern analysis

To detect genes that are differentially expressed under hypoxia and normoxia, RSEM and edgeR were used with a false discovery rate (FDR) threshold of ≤0.05 and fold change of ≥2. For *L. mandarinus*, 695 differentially expressed genes (DEGs) were identified from a total of 69,611 unigenes, of which 425 were upregulated and 270 were downregulated in the hypoxic brain relative to the normoxic brain (Additional file 1: Figure S1). Of the 695 DEGs, 289 were annotated in at least one of the following databases: Nr (*n* = 270), Nt (*n* = 208), Swissprot (*n* = 239), and KEGG (*n* = 170). Of the 270 DEGs annotated in the Nr database, 47 were described as “hypothetical” in the GenBank database and were therefore excluded from further analysis. Ultimately, 223 DEGs that were annotated in Nr were screened, of which 110 were upregulated and 113 were downregulated (Additional file 2: Table S5).

For *L. brandtii*, there were only 158 DEGs among a total 69,360 unigenes, with 104 upregulated and 54 downregulated in the hypoxic brain relative to the normoxic brain (Additional file 1: Figure S1). Of the 158 DEGs, 110 were annotated in at least one of the following databases: Nr (*n* = 103), Nt (*n* = 76), Swissprot (*n* = 93), and KEGG (*n* = 73). Of the 103 DEGs annotated in the Nr database, 14 were described as “hypothetical” in the GenBank database and were therefore excluded from further analysis. Ultimately, 89 DEGs that were annotated in Nr were screened, of which 49 were upregulated and 40 were downregulated (Additional file 2: Table S5).

### DEG functional enrichment analysis

**Fig. 2:**
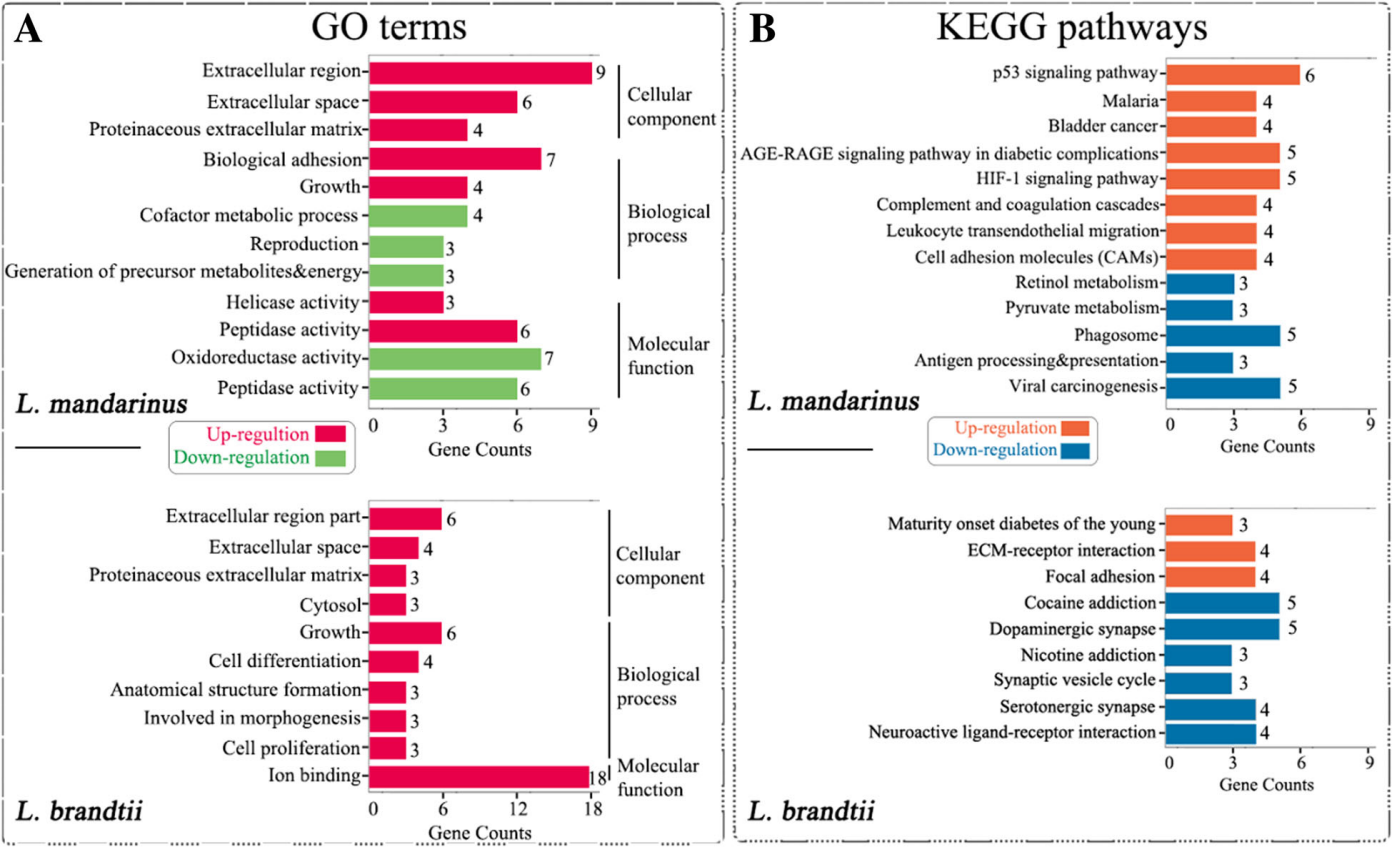
**a** GO terms and **b** KEGG pathways significantly enriched for up- and downregulated DEGs in *L. mandarinus* and *L. brandtii*

The GO enrichment analysis indicated that the upregulated DEGs of *L. mandarinus* were enriched in seven GO terms in three categories—i.e., biological process (*n* = 2), cellular component (*n* = 3), and molecular function (*n* = 2). There were also five enriched GO terms among the downregulated DEGs in *L. mandarinus*. For *L. brandtii*, all the DEGs were upregulated and were enriched in 10 GO terms. (Fig. 2a and Additional file 1: Table S6).

The GO categories enriched for the upregulated DEGs of *L. mandarinus* represented biological functions such as endothelial cell proliferation (Extracellular region and growth), cell migration, cell differentiation (Biological adhesion), gene expression (Helicase activity), and energy harvesting (Peptidase activity); whereas enriched GO terms for downregulated DEGs included coenzymes (Generation of precursor metabolites and energy), oxidoreductases (Oxidoreductase activity), and proteases (Peptidase activity) related to aerobic reactions. Notably, peptidase activity was found to be enriched among DEGs that were up- and downregulated in *L. mandarinus* (Additional file 1: Table S7).

Three GO terms were shared by *L. brandtii* and *L. mandarinus*, whereas six differed between the two species that were related to vascular endothelial cell proliferation (Cell proliferation), cell differentiation (Cell differentiation), and cell migration (Anatomical structure formation involved in morphogenesis).

### DEG pathway analysis

To clarify the relationships between the DEGs, we mapped the genes in the KEGG pathway database and performed enrichment analysis with Fisher’s exact test. Pathways with fewer than three genes were discarded, and those with both *P* value < 0.05 and FDR < 0.05 were selected as enriched pathways. We identified eight enriched pathways for upregulated and five for downregulated DEGs in *L. mandarinus*, and three pathways for upregulated and six for downregulated DEGs in *L. brandtii* (Fig. 2b and Additional file 1: Table S8).

Among the eight enriched pathways for upregulated DEGs in *L. mandarinus*, the major functions were angiogenesis, cell proliferation, and apoptosis (AGE-RAGE signaling pathway in diabetic complications, Bladder cancer, and HIF-1 signaling). Some were involved in the regulation of cell transendothelial migration (Leukocyte transendothelial migration and Cell adhesion molecule), inhibition of angiogenesis, or induction of cell cycle arrest, cell repair, and apoptosis (p53 signaling). The DEGs in the malaria pathway included two subunits of heme (HBA and HBB). An increase in heme can significantly increase the O_2_-carrying capacity of red blood cells. Of the five enriched pathways for downregulated DEGs, most were related to the immune response and shared a common feature of O_2_ consumption.

In *L. brandtii*, the pathways associated with the three upregulated DEGs were linked to cell survival (Maturity onset diabetes of the young), extracellular matrix (ECM)-related vascular endothelial cell proliferation (ECM-receptor interaction), and neuroprotection (Focal adhesion). However, downregulated DEGs were enriched in six KEGG pathways that were related to synaptic transmission in neurons.

### Comparative analysis of unique differentially expressed genes

**Fig. 3:**
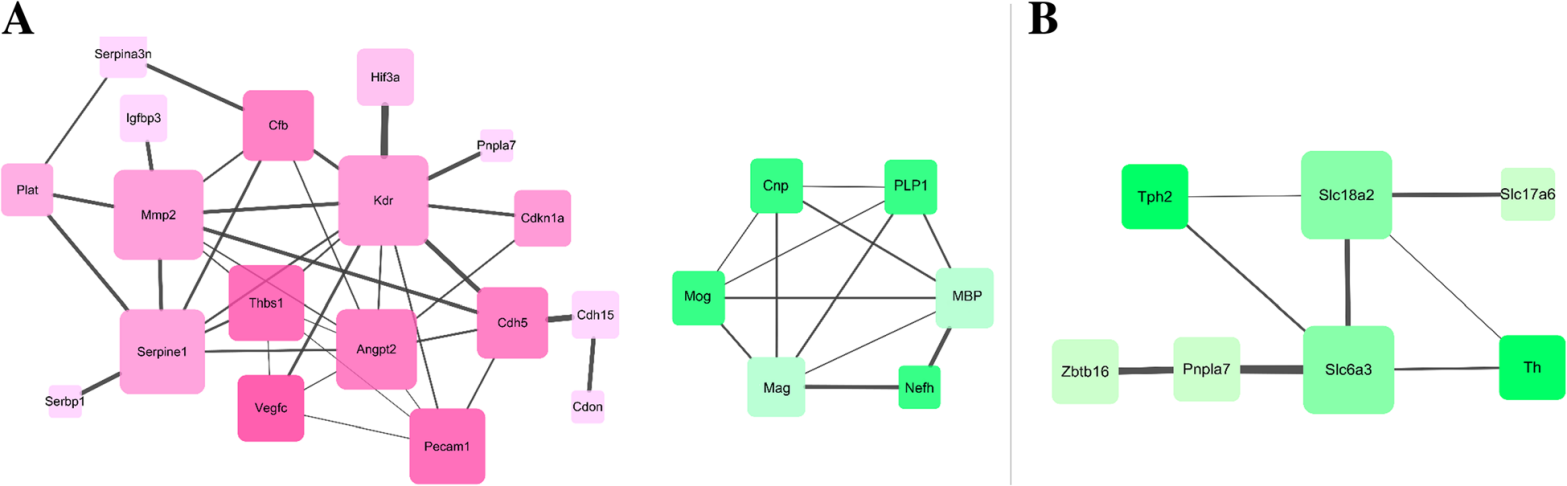
Protein interaction network for specific DEGs in *L. mandarinus* (**a**) and *L. brandtii* (**b**) brain under chronic hypoxia

To further analyze the similarities and differences between the *L. mandarinus* and *L. brandtii* hypoxia adaptations, we removed DEGs shared by the two species and constructed protein interaction networks for their unique DEGs (Fig. 3). Among the chronic hypoxia-specific differentially expressed genes in *L. mandarinus*, the most prominent cores were *MMP2*, *THBS1, SERPINE1*, and *VEGFR-2*, which are responsible for angiogenesis regulation /inhibition. It is worth noting that MBP is a marker protein of cranial nerve damage that is regulated by multiple proteins. The down-regulation of MBP as well as the expression of other interacting proteins indicates that neurons in *L. mandarinus* are less prone to hypoxia-induced injury. The most significant cores of the chronic hypoxia-specific differentially expressed gene protein interaction network in *L. brandtii* are dopamine synthesis and transport regulation-related proteins. The reduction of these proteins leads to decreased neurogenic excitability and decreased activity in *L. brandtii*.

### Validation of DEGs by reverse-transcription quantitative (RT-q)PCR analysis

**Fig. 4:**
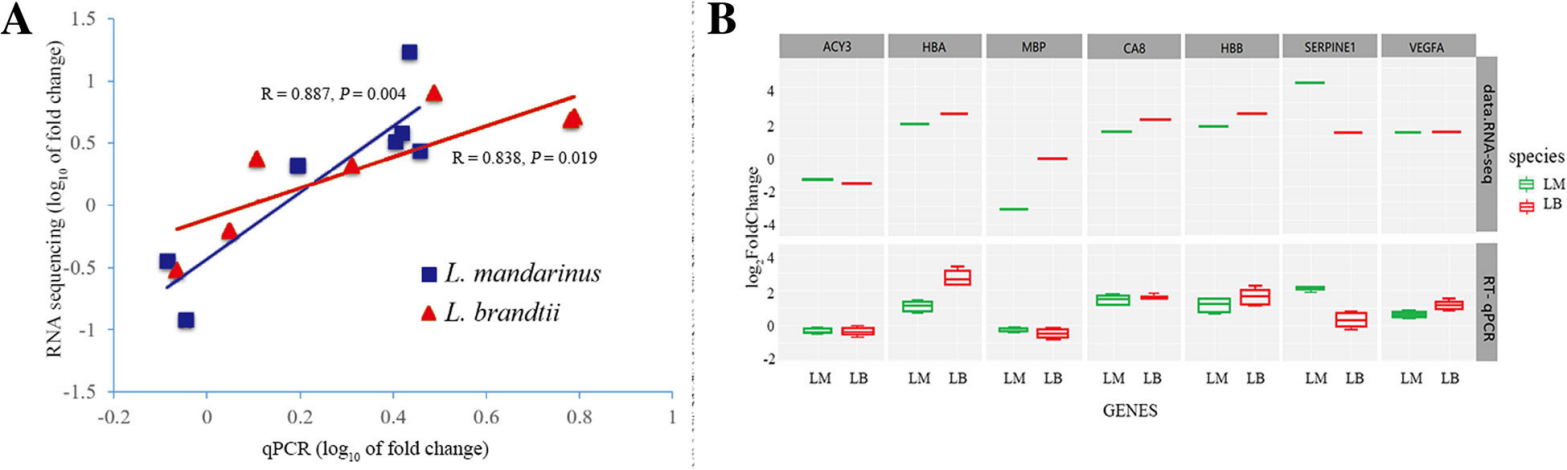
Validation of RNA sequencing results by RT-qPCR. **a** Correlations between gene expression levels measured by RT-qPCR and RNA-Seq methods. **b** Comparison of RNA-Seq log_2_FoldChange read counts with log_2_FoldChange RT-qPCR copy numbers. The upper panel shows the RNA-Seq read counts (log_2_FoldChange) for seven genes, of which five are upregulated in chronic hypoxia vs. normoxia in *L. mandarinus* and *L. brandtii* brain and two are downregulated. The lower panel shows log_2_FoldChange by RT-qPCR copy numbers for the same genes in chronic hypoxia vs. normoxia in *L. mandarinus* and *L. brandtii* brain samples. (LM: *L. mandarinus*; LB: *L. brandtii*)

To validate the expression data obtained from RNA sequencing (RNA-Seq), seven genes with different expression patterns between *L. mandarinus* and *L. brandtii* were randomly selected to perform qPCR. Among the seven selected genes, five genes were up-regulated and two were down-regulated in *L. mandarinus*, compared with *L. brandtii*. The results showed a strong correlation between the data of RNA-Seq and those of qPCR (*R* = 0.887, *P* = 0.004 for *L. mandarinus* and *R* = 0.838, *P* = 0.019 for *L. brandtii*; Fig. 4a), indicating that the data from our transcriptome analysis were reliable. The expression profile of the seven genes validated via RT-qRCR is shown in Fig. 4b.

## Discussion

**Fig. 5:**
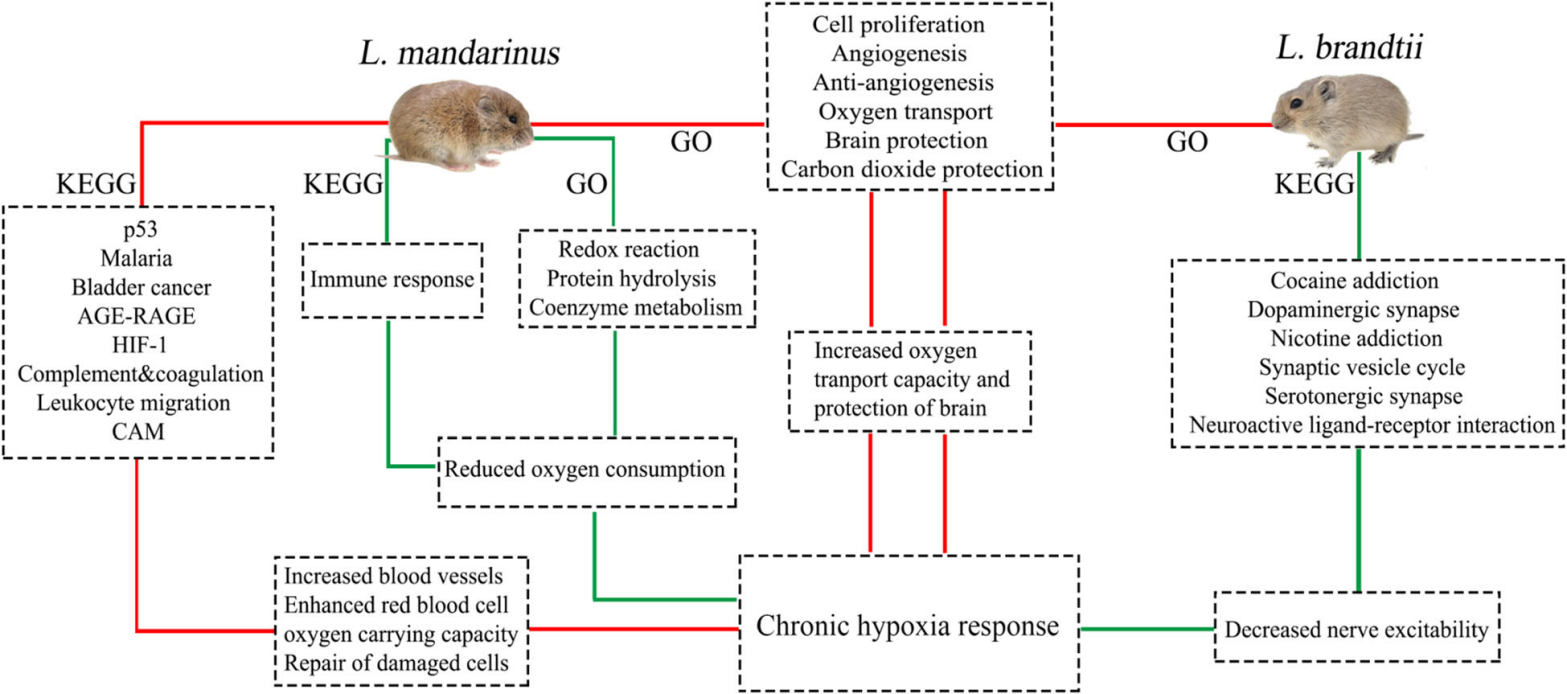
Presumed responses of *L. mandarinus* and *L. brandtii* to chronic hypoxia. Red and green lines indicate up- and downregulated gene enrichment, respectively. The images of voles were photographed in the animal laboratory of the school of life sciences, Zhengzhou University

In this study, we carried out a comparative transcriptome analysis between *L. mandarinus* and *L. brandtii* whole brain tissues exposed to chronic hypoxia vs. normoxia. Our results reveal that similar GO terms were enriched for upregulated DEGs in *L. mandarinus* and *L. brandtii* under chronic hypoxia; these were associated with endothelial cell proliferation, cell migration, gene expression, angiogenesis, angiogenesis inhibition, energy acquisition, O_2_ transport, neuroprotection, and protection from carbon dioxide [13, 19] (Fig. 5 and Additional file 1: Table S6). This suggests that both *L. mandarinus* and *L. brandtii* respond to chronic hypoxic stress by increasing the number of blood vessels, improving blood flow, and enhancing O_2_ transport capacity. Additionally, in *L. mandarinus*, enriched GO terms for downregulated DEGs were related to cofactors and enzymes such as NADH dehydrogenase, NAD-dependent malic enzyme, prenylcysteine oxidase, and peroxisomal sarcosine oxidase (Fig. 2a and Additional file 2:Table S5), which are also enriched in the Upper Galilee Mountains blind mole-rat *Spalax galili* [20]. A previous study of *S. galili* indicated that tight control of angiogenesis may be a novel mechanism of hypoxia tolerance in subterranean rodents [14]. Our results demonstrate that as O_2_ supply decreases, *L. mandarinus* and *L. brandtii* reduce O_2_ consumption—the former, by suppressing O_2_-consuming reactions such as redox reactions, proteolysis, and coenzyme metabolism—and/or redirect O_2_ usage. Besides, hypoxia-tolerance studies of heterothermic *Heterocephalus glaber* (the naked mole-rat) found that it can endure tissue hypoxic conditions by actively reducing brain oxygen consumption, and it was suggested that its protective strategies include modulation of immune response, thrombolysis, antioxidant defense, and activation or inactivation of pre-existing proteins [21, 22]. These results suggest that reducing brain oxygen consumption may be a common way for subterranean rodents to adapt to hypoxia conditions.

Peptidase activity was enriched among up- and downregulated DEGs in *L. mandarinus* (Fig. 2a and Additional file 1: Table S7). The upregulated group showed the activation of genes such as A disintegrin and metalloproteinase with thrombospondin motifs (ADAMTS)1 and ADAMTS9, which are involved in angiogenesis inhibition. In addition, complement factor B (CFB) and MMP2 are important for angiogenesis [23]; plasminogen activator tissue type (PLAT) contributes to thrombolysis [24], and autophagy 4C (ATG4C) is related to autophagy [25, 26]. Among the downregulated DEGs, transmembrane protease serine 5 (TMPRSS5) and chymotrypsin-like elastase family member 1 (CELA1) are serine proteases that activate the complement system and induce blood coagulation; fibrinogen-like protein 2 (FGL2) and coagulation factor IX (F9) are highly expressed in vascular endothelial cells and accelerate blood coagulation; and ADAMTS4 and ADAMTS2 are collagen precursors that are involved in coagulation and cell support.

Enriched pathways for upregulated DEGs in *L. mandarinus* were associated with angiogenesis (HIF-1 signaling, bladder cancer, and AGE-RAGE signaling pathway in diabetic complications), transendothelial migration (leukocyte transendothelial migration and cell adhesion molecules), angiogenesis inhibition, cell cycle arrest for cell repair, and apoptosis (p53 signaling pathway). These pathways may be involved in increasing blood vessels, enhancing red blood cell O_2_ transport capacity, and repairing damaged cells. Enriched pathways for downregulated DEGs in *L. mandarinus* were mainly related to aerobic metabolism and immune response, which could supply additional O_2_ for cell survival (Fig. 5).

**Fig. 6:**
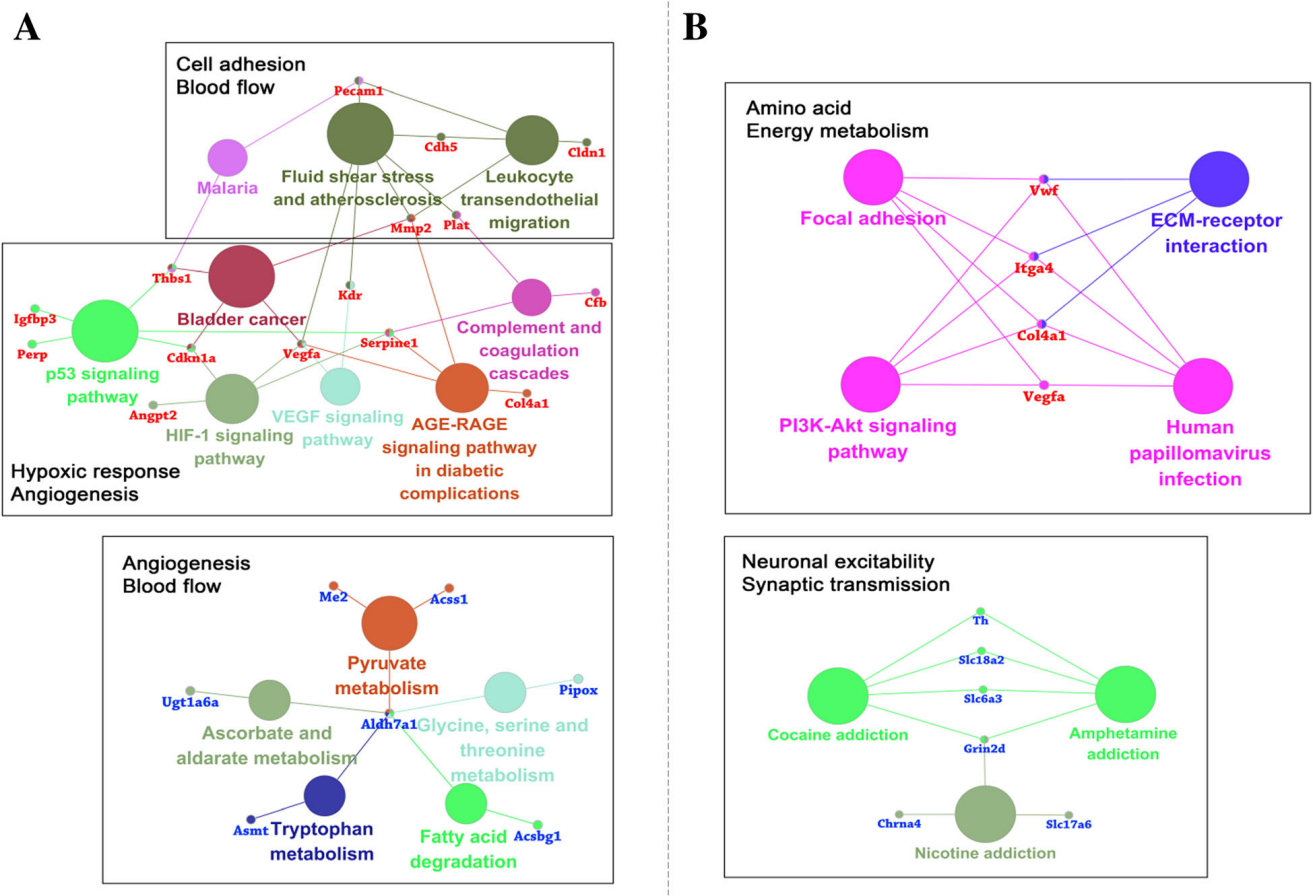
Gene-pathway networks for DEGs in (**a**) *L. mandarinus* and (**b**) *L. brandtii* under chronic hypoxia. Nodes (circles) represent DEGs and enriched pathways for DEGs, respectively. Genes indicated in red and blue are up- and downregulated, respectively. Lines between nodes represent connections between genes and pathways in the network

The metabolic pathway network in *L. mandarinus* can be roughly divided into three categories (Fig. 6a). The first includes pathways and genes related to hypoxia stress, angiogenesis and its inhibition, apoptosis, and cell repair; and the second involves genes that are associated with the ECM and regulate blood flow. Most of these genes were upregulated. Interestingly, the results for the first category were consistent with research findings for *S. galili* compared with *Spalax judaei*. Avivi et al. uncovered that *S. galili* exhibits species-specific responses to hypoxic stress via numerous genes involved in angiogenesis, apoptosis, and oxidative stress management [27]. In contrast, the third category comprises downregulated genes and pathways involved in amino acid synthesis and energy metabolism. Other studies that compared transcript abundance in *Spalax* vs. rat whole brain tissues showed that down-regulated genes in *Spalax* were significantly associated with carbohydrate metabolism, lipid metabolism, redox metabolism, mitochondria inner membrane activity, and oxidative phosphorylation [20]. These findings on the hypoxic adaptation of *Spalax* are similar to those of our third category in *L. mandarinus*. Genes with more connections in the gene-metabolism network such as *VEGFA*, matrix metallopeptidase (*MMP*)*2* [28], serpin peptidase inhibitor [29], and thrombospondin 1 are associated with angiogenesis and its inhibition; their upregulation indicates that angiogenesis in *L. mandarinus* is in a state of dynamic equilibrium under chronic hypoxia.

Upregulated pathways unique to *L. brandtii* hypoxic brain were involved in ECM-related endothelium cell proliferation and cell survival (ECM-receptor interaction and focal adhesion) as well as neuroprotection (maturity onset diabetes of the young) [30]. It is worth noting that all six downregulated pathways in *L. brandtii* were related to synaptic transmission, suggesting that *L. brandtii* reduces its activity level in order to reduce O_2_ consumption under hypoxic conditions (Fig. 5). The DEG-metabolic pathway network of *L. brandtii* can be divided into two parts according to gene function (Fig. 6b). The first part mainly includes ECM and adhesion-related genes such as *Vwf* (Von Willebrand factor) [31], *COL4A1* (collagen, type IV, alpha 1), *ITGA4* (integrin alpha 4), and *VEGFA*. The increased expression of these genes is linked to vascular endothelial cell proliferation and angiogenesis [32]. The second part of the metabolic network is related to synaptic transmission. Key genes in the network including *TH* (tyrosine hydroxylase), *GRIN2D* (glutamate ionotropic receptor NMDA type subunit 2D), solute carrier family (*SLC*)*6A3* (neurotransmitter transporter, dopamine), and *SLC18A2* were downregulated. These four genes are involved in the production, transport, and synaptic transmission of the neurotransmitter dopamine; their downregulation decreases dopamine release, thereby reducing neuronal excitability [33].

Differences in gene expression between the two species of vole in this study under chronic hypoxia are mainly determined by their distinct life histories. *L. mandarinus* lives in an underground tunnel system for most of its life; during summer when there is abundant rainfall, tunnels can collapse as the earth becomes wet. To repair the tunnel under hypoxic conditions, *L. mandarinus* maintains a high blood pressure and neuronal excitability and has evolved an enhanced O_2_-transport capacity as well as mechanisms to reduce O_2_ consumption and protect the brain from hypoxic injury.

In contrast, *L. brandtii* also burrows in tunnels but this is mainly used for rest; O_2_-consuming activities such as mating, feeding, and excavation are performed above ground. A hypoxic environment develops when snowfall covers the ground in winter. During this time, *L. brandtii* performs few activities in the tunnel and there is little risk of tunnel collapse. This explains the relatively passive response of *L. brandtii* to chronic hypoxia, including reductions in blood pressure and nerve excitability.

Presently, research on activities related to hypoxia, such as human life on the plateau or diving, has received much attention. Our findings may shed new light on human adaptation to hypoxia conditions. In fact, many people, including Tibetans, Andeans, and Ethiopians are currently living in long-term hypoxic plateau environments [34]. However, the mechanisms of high-altitude adaptation in different human populations are distinct and complex. Mounting evidence suggests that the observed physiological adaptations are controlled by interactions among multiple genes, especially those that are part of the HIF pathway. Tibetans inhale more air with each breath and breathe more rapidly than either sea-level inhabitants or Andeans [35]. In addition, they have high levels of NO (nitric oxide) in their blood when compared to low land dwellers, and this probably aids the dilation of their blood vessels to enhance blood circulation [36]. The patterns of genetic adaptation among Andeans are largely distinct from those of Tibetans. For instance, the lack of significant associations between *EPAS1* or *EGLN1* SNP genotypes and hemoglobin concentration is characteristic of Tibetans [37]. For Ethiopians, adaptation to hypoxia involves several candidate genes including *CBARA1, VAV3, ARNT2,* and *THRB* [38]. *THRB* and *ARNT2* are known to play a role in the HIF-1 pathway, a pathway implicated in a previous study conducted in Tibetan and Andean populations [39]. In addition to facing hypoxic conditions in the living environment, some special human activities, such as diving, frequently expose certain individuals to hypoxic conditions. Early studies of diving populations suggested that they were adapted to hypoxic conditions via bradycardia and peripheral vasoconstriction, which lower oxygen consumption and selectively redistribute blood flow to the organs most sensitive to hypoxia [40, 41]. A recent comparative genomic study revealed natural selection of genetic variants in *PDE10A* in the “Sea Nomads” (The indigenous Bajau people) and suggested that mutations in this gene led to an increase in spleen size, thereby providing the people with a larger reservoir of oxygenated red blood cells [42].

Compared with human hypoxia adaptation studies, although some physiological or pathway changes, such as adjustment of angiogenesis and contraction or interaction of some genes in the HIF signaling pathway or other pathways (VEGF signaling pathway), were similar to the results of our studies on *L. mandarinus*, the genes involved in these pathways differed between humans and *L. mandarinus*. These may be related to the large differences in hypoxia conditions between humans and subterranean rodents.

## Conclusions

Long-term existence in underground tunnels has enabled *L. mandarinus* to better adapt to hypoxia than the closely related species *L. brandtii*. At 10% O_2_, *L. mandarinus* actively adapts its physiological functions by increasing O_2_ transport capacity and reducing O_2_ consumption, whereas *L. brandtii* reacts passively by decreasing its activity. These results provide insight into mechanisms of hypoxia adaptation in subterranean rodents that may be applicable to humans living in high-altitude or other O_2_-poor environments.

## Methods

### Animals and hypoxia treatment

*L. mandarinus* was trapped live from croplands in Xinzheng, Henan, China (N 34°52′, E 113°85′), and *L. brandtii* was imported from the Chinese Academy of Agricultural Science. The animals were maintained individually in polycarbonate cages (37 × 26 × 17 cm) in the laboratory on a 14:10-h light/dark cycle at a temperature of 20 °C–24 °C.

To mimic chronic hypoxic stress, 12 healthy adult male voles (3 months of age; *n* = 6 of each species) were randomly divided into the hypoxia (10% O_2_ for 48) and normoxia (20.9% O_2_ for 48 h) groups. A DS-II hyperbaric cabin (Huaxin Hyperbaric Cabin, Weifang, China) was used to simulate chronic hypoxia. O_2_ level in the cabin was maintained at a constant level by balancing the flow rate of O_2_ and N_2_, and was monitored with an Oxygen analyzer (Talantek, Beijing, China). A bottle of sodium hydroxide was placed in the cabin to absorb the CO_2_ released by the animals. Once the treatment was completed, the animals were immediately sacrificed with an overdose of pentobarbital sodium. The brain was removed and immediately frozen in liquid nitrogen and stored at − 80 °C until use.

### RNA extraction, cDNA library preparation, and RNA-Seq

Experimental procedures including sample preparation and RNA-Seq were performed according to standard protocols. Total RNA was extracted from each sample using TRIzol reagent (Invitrogen, Carlsbad, CA, USA) according to the manufacturer’s instructions. The RNA was treated with RNase-free DNase I (Takara Bio, Dalian, China) to remove residual DNA. RNA integrity was verified by agarose gel electrophoresis (1.2%), and RNA concentration was measured with an Agilent 2100 Bioanalyzer (Agilent Technologies, Santa Clara, CA, USA).

High-quality RNA samples were sent to Biomarker Technologies Corp. (Beijing, China) for cDNA library construction and sequencing, with mRNAs purified through interaction of the poly(A) tails and magnetic oligo(dT) beads. RNA-Seq libraries were generated using the TruSeq RNA Sample Prep kit (Illumina, San Diego, CA, USA) and multiplexing primers according to the manufacturer’s protocol. The cDNA library was constructed with average inserts of 250 bp (150–250 bp) by non-stranded library preparation. The cDNA was purified using a QiaQuick PCR extraction kit (Qiagen, Hilden, Germany). The short cDNA fragments were subjected to end repair, adapter ligation, and agarose gel electrophoresis filtration, and suitable fragments were selected as templates for PCR amplification. Sequencing was performed via a paired-end 125-cycle rapid run on the Illumina HiSeq2500 system.

### Read filtering and sequence assembly

High-quality clean reads were obtained by removing adaptor sequences, duplicated sequences, and ambiguous (‘N’) and low-quality reads. Transcriptomes of the two species were separately assembled de novo using the short-reads assembly program Trinity [43]. After assembly, the TIGR Gene Indices clustering tools were used to cluster and remove redundant transcripts [44]. The longest transcripts were considered as unigenes after removing redundancies, and these were subjected to downstream functional annotation and coding sequence (CDS) prediction [45].

### Functional annotation

The unigenes of *L. mandarinus* and *L. brandtii* were compared using BLASTX against the Nr, KEGG [46], GO [47], the Eukaryotic Orthologous Groups (KOG) [48], and Swiss-Prot [49] databases (*E*-value ≤1e^− 5^) to retrieve protein functional annotations based on sequence similarity. Gene names were assigned based on the best BLAST hit. High-priority databases (followed by Nr, Swiss-Prot, and KEGG) were selected to determine the direction of unigene sequences. Sequences showing the best alignment were used to predict the CDSs, and TransDecoder (Find Coding Regions Within Transcripts, https://transdecoder.github.io/) was used to identify candidate coding regions within transcript sequences. CDSs were translated into amino sequences using the standard codon Table. GO terms including molecular function, biological process, and cellular component categories were assigned to each sequence using Blast2GO software with an E-value threshold of 1e^− 6^ for further functional categorization [50]. The distribution of the GO functional classifications of unigenes was plotted using the OmicShare tool (http://www.omicshare.com/tools). The unigenes were also aligned to the Cluster of Orthologous Groups/KOG database to predict and classify possible functions. KOBAS 2.0 software [51] (http://kobas.cbi.pku.edu.cn/) was used to assign unigenes to KEGG pathway annotations and analyze metabolic pathways.

### Identification of DEGs

The clean reads of the *L. mandarinus* and *L. brandtii* sequences were mapped with their respective unigenes by RSEM package [52]. The Fragments Per Kilobase of exon per Million mapped fragments (FPKM) was used to eliminate the influence of different gene lengths and sequencing levels on the calculation of gene expression, and FPKM values were directly used to compare gene expression differences between samples. The edgeR package [53] (http://bioconductor.org/packages/release/bioc/html/edgeR.html) was used to obtain the base mean value for identifying DEGs. To correct for multiple testing, the FDR was calculated to adjust the *P* value threshold. Transcripts with an FDR ≤ 0.05 and a minimum of 2-fold difference in expression (|log_2_ ratio| ≥ 1) were considered as thresholds for the significance of gene expression differences between two groups. In addition, information for DEGs was collected from unigene annotations, and these genes were subjected to GO and KEGG significant enrichment analyses to identify biological functions and metabolic pathways involving these genes.

### Validation of RNA-Seq results by RT-qPCR

To validate the reliability of DEGs identified by RNA-Seq, the mRNA expression levels of seven selected genes were measured by RT-qPCR using the same samples. Primers were designed using Primer-BLAST; the sequences are shown in Additional file 1: Table S1. All primer sets yielded a single peak in the dissociation curves with an amplification efficiency of approximately 1.0. Three technical replicates were prepared for each gene in 96-well plates and amplification was performed on a LightCycler® 480 Instrument II (Roche Diagnostics, Mannheim, Germany). Relative gene expression levels were normalized to that of the internal reference gene β-actin and were calculated with the 2^−ΔΔCt^ method. Correlation analysis in SPSS v.19.0 (SPSS Inc., Chicago, IL, USA) was used to evaluate the concordance between RT-qPCR results and RNA-Seq data. Differences were defined as significant at *P* < 0.05 and highly significant at *P* < 0.01 (Welch’s t-test).

## Additional files

Additional file 1: **Table S1.** RT-qPCR primers for validation of RNA-Seq data. **Table S2.** Illumina sequencing data for analyzed samples. **Table S3.** Length distribution of assembled unigenes. **Table S4.** Functional annotation results for *L. mandarinus* and *L. brandtii* transcriptomes. **Table S6.** GO terms significant enriched for up- and downregulated DEGs in *L. mandarinus* and *L. brandtii*. **Table S7.** Genes associated with the GO term “peptidase activity” among up- and downregulated DEGs in *L. mandarinus*. **Table S8.** KEGG pathways enriched for up- and downregulated DEGs in *L. mandarinus* and *L. brandtii* under acute hypoxia. **Figure S1.** DEGs in the brain of *L. mandarinus* and *L. brandtii* under chronic hypoxia vs. normoxia. FC, fold change; FDR, false discovery rate. Red, blue, and green dots represent up- and downregulated and unchanged genes, respectively. (DOCX 481 kb)

Additional file 2: **Table S5.** DEGs for *L. mandarinus* and *L. brandtii*. (XLSX 48 kb)

## Abbreviations

DEGs: Differentially expressed genes
GO: Gene Ontology
KEGG: Kyoto encyclopedia of genes and genomes
*L. brandtii*: *Lasiopodomys brandtii*
*L. mandarinus*: *Lasiopodomys mandarinus*
RT-qPCR: Reverse-transcription quantitative PCR
