# Supplementary material for: Differential responses of Lasiopodomys mandarinus and Lasiopodomys brandtii to chronic hypoxia: a cross-species brain transcriptome analysis
Source: BMC Genomics. 2018 Dec 11;19:901. doi: 10.1186/s12864-018-5318-1 (PMC6290494; doi:10.1186/s12864-018-5318-1)

**Table S1.** RT-qPCR primers for validation of RNA-Seq data

| Gene | Primer-Forward | Primer-Reverse | efficiency |
| --- | --- | --- | --- |
| ACY3 | CACAGACCAGGCTTTCGACT | ACAGGTGCAGGTTGAAGGAG | 1.36 |
| HBA | CGGCAAGAAAGTCGCTGATG | AAGTTGACAGGGTCCACACG | 1.06 |
| HBB | GCCTGAAACACCTGGACAAC | TGGGACAGCACAATCACGAT | 1.16 |
| VEGFA | ATGAGGCCCTGGAATGTGTG | GCTGGCTTTGGTGAGGTTTG | 1.44 |
| CA1 | GCGGGATTACTGGGTCTACG | GTCCTCAGCCTTCGGAACTC | 1.39 |
| SEPRINE1 | AGCAAGCGGGAAGAAGAGTC | CAGGGTGAGGCAAGCTAGTG | 0.78 |
| MBP | ATGCCCATGTGCTGAGTCAA | GCAGGTCCTTCACGATGTCT | 1.35 |
| β-actin | GTCGTACCACTGGCATTGTG | CCATCTCTTGCTCGAAGTCC | 1.05 |

**Table S2.** Illumina sequencing data for analyzed samples

| Samples | Total Raw Reads Number | Total Clean Reads Number | Total Clean  Bases Number | Clean Reads  GC Content (%) | Clean Reads Q30 (%) | Clean Reads Ratio (%) |
| --- | --- | --- | --- | --- | --- | --- |
| CK1-LM | 28,737,984 | 26,102,782 | 6,574,275,667 | 48.81 | 91.71 | 90.83 |
| CK2-LM | 28,679,082 | 26,042,739 | 6,559,207,285 | 49.01 | 91.31 | 90.81 |
| CK3-LM | 34,655,748 | 31,035,722 | 7,814,838,065 | 49.14 | 91.03 | 89.55 |
| Hyp1-LM | 29,371,440 | 26,842,471 | 6,762,197,929 | 49.13 | 91.50 | 91.39 |
| Hyp2-LM | 30,978,734 | 28,519,975 | 7,185,613,435 | 49.27 | 91.60 | 92.06 |
| Hyp3-LM | 30,563,206 | 27,987,742 | 7,051,285,859 | 49.06 | 91.57 | 91.57 |
| CK1-LB | 39,107,546 | 35,420,510 | 8,920,764,050 | 50.01 | 91.43 | 90.57 |
| CK2-LB | 35,050,234 | 32,039,776 | 8,071,534,141 | 49.46 | 91.43 | 91.41 |
| CK3-LB | 32,946,493 | 29,979,937 | 7,551,480,911 | 49.15 | 91.34 | 91.00 |
| Hyp1-LB | 30,080,266 | 27,558,229 | 6,942,675,804 | 49.02 | 91.57 | 91.62 |
| Hyp2-LB | 33,176,176 | 30,222,063 | 7,614,633,673 | 49.24 | 90.90 | 91.10 |
| Hyp3-LB | 36,253,900 | 33,273,369 | 8,383,400,849 | 49.23 | 91.63 | 91.78 |
| Total | 389,600,809 | 355,025,315 | 89,431,907,668 |  |  |  |

**Sample**: Sample name; **Total Raw Reads** **Number**: The reads amount before filtering; **Total Clean Reads Number**: The reads amount after filtering; **Total Clean Bases Number**: The total base amount after filtering; **GC(%)**: the percentage of G and C bases in all Clean reads; **Clean Reads Q30(%)**: The rate of bases which quality is greater than 30 value in clean reads; **Clean Reads Ratio(%)**: The ratio of the amount of clean reads.

**Table S3.** Length distribution and quality metrics of unigenes from the *L. mandarinus* and *L. brandtii*

| Length Range(bp) | *L. mandarinus* | *L. brandtii* |
| --- | --- | --- |
| 200- 300 | 24598 | 23952 |
| 300- 500 | 17275 | 17340 |
| 500-1000 | 11557 | 11932 |
| 1000-2000 | 7188 | 7255 |
| 2000+ | 8993 | 8881 |
| Total Number  Total Length(bp)  Mean Length(bp)  N50(bp)  GC(%) | 69,611  65,744,857  944.46  2214  49.18 | 69,360  67,904,229  974.89  2306  49.42 |

**Length Range**: Length distribution of assembled unigenes; **Total Number**: The total number of Unigenes; **Total Length**: The read length of Unigenes; **Mean Length**: The average length of Unigenes; **N50**: The N50 length is used to determine the assembly continuity, the higher the better. N50 is a weighted median statistic that 50% of the total length is contained in transcripts that are equal to or larger than this value. **GC(%)**: the percentage of G and C bases in all Unigenes.

**Table S4.** Functional annotation results for *L. mandarinus* and *L. brandtii* transcriptomes

| Database | *L. mandarinus* | *L. brandtii* |
| --- | --- | --- |
| GO | 10593 | 9838 |
| KEGG | 11531 | 10673 |
| KOG | 13105 | 12493 |
| Swissprot | 17390 | 16504 |
| Nr | 20011 | 19120 |
| All | 20172 | 19215 |

**Table S6.** GO terms significant enriched for up- and downregulated DEGs in *L. mandarinus* and *L. brandtii*

| ***L. mandarinus*** | | | ***L. brandtii*** | | |
| --- | --- | --- | --- | --- | --- |
| **Up-regulated** | | | **Up-regulated** | | |
| **GO terms** | **GO ID** | ***P* value** | **GO terms** | **GO ID** | ***P value*** |
| extracellular region | 0005576 | <0.001 | extracellular region part | 0044421 | <0.001 |
| extracellular space | 0005615 | <0.001 | proteinaceous extracellular matrix | 0005578 | 0.001 |
| proteinaceous extracellular matrix | 0005578 | 0.017 | extracellular space | 0005615 | 0.007 |
| biological adhesion | 0022610 | 0.010 | cytosol | 0005829 | 0.013 |
| growth | 0040007 | 0.030 | cell differentiation | 0030154 | 0.001 |
| helicase activity | 0004386 | 0.009 | anatomical structure formation involved in morphogenesis | 0048646 | 0.001 |
| peptidase activity | 0008233 | 0.033 | growth | 0040007 | 0.016 |
|  |  |  | cell proliferation | 0008283 | 0.031 |
|  |  |  | ion binding | 0043167 | 0.063 |
| **Down-regulated** | | | **Down-regulated** | | |
| cofactor metabolic process | 0051186 | 0.012 |  |  |  |
| reproduction | 0000003 | 0.027 |  |  |  |
| generation of precursor metabolites and energy | 0006091 | 0.048 |  |  |  |
| oxidoreductase activity | 0016491 | 0.009 |  |  |  |
| peptidase activity | 0008233 | 0.011 |  |  |  |

Terms with P < 0.05 are shown, with a Benjamini-Hochberg correction or false discovery rate. BP, biological process; CC, cellular component; MF, molecular function.

**Table S7.** Genes associated with the GO term “peptidase activity” among up- and downregulated DEGs in *L. mandarinus*

| up-regulated DEGs | down-regulated DEGs |
| --- | --- |
| ADAMTS 1 | TMPRSS5 |
| ADAMTS 9 | F 9 |
| CFB | CELA1 |
| MMP2 | FGL2 |
| PLAT | ADAMTS 4 |
| ATG4C | ADAMTS 2 |

**Table S8.** KEGG pathways enriched for up- and downregulated DEGs in *L. mandarinus* and *L. brandtii* under acute hypoxia

| ***L. mandarinus*** | | | ***L. brandtii*** | | |
| --- | --- | --- | --- | --- | --- |
| **Up-regulated** | | | **Up-regulated** | | |
| **Pathway** | **Map ID** | ***P* value** | **Pathway** | **Map ID** | ***P value*** |
| p53 signaling pathway | 04115 | <0.001 | Maturity onset diabetes of the young | 04950 | <0.001 |
| Malaria | 05144 | <0.001 | ECM-receptor interaction | 04512 | 0.001 |
| Bladder cancer | 05219 | <0.001 | Focal adhesion | 04510 | 0.011 |
| AGE-RAGE signaling pathway in diabetic complications | 04933 | 0.003 |  |  |  |
| HIF-1 signaling pathway | 04066 | 0.003 |  |  |  |
| Complement and coagulation cascades | 04610 | 0.003 |  |  |  |
| Leukocyte transendothelial migration | 04670 | 0.015 |  |  |  |
| Cell adhesion molecules (CAMs) | 04514 | 0.016 |  |  |  |
|  |  |  |  |  |  |
| **Down-regulated** | | | **Down-regulated** | | |
| Retinol metabolism | 00830 | <0.001 | Cocaine addiction | 05030 | <0.001 |
| Pyruvate metabolism | 00620 | 0.001 | Dopaminergic synapse | 04728 | 0.000 |
| Phagosome | 04145 | 0.002 | Nicotine addiction | 05033 | 0.001 |
| Antigen processing and presentation | 04612 | 0.006 | Synaptic vesicle cycle | 04721 | 0.002 |
| Viral carcinogenesis | 05203 | 0.012 | Serotonergic synapse | 04726 | 0.002 |
|  |  |  | Neuroactive ligand-receptor interaction | 04080 | 0.010 |

Pathways with FDR < 0.05 are shown.

**Figure S1.** DEGs in the brain of *L. mandarinus* and *L. brandtii* under chronic hypoxia vs. normoxia. FC, fold change; FDR, false discovery rate. Red, blue, and green dots represent up- and downregulated and unchanged genes, respectively.


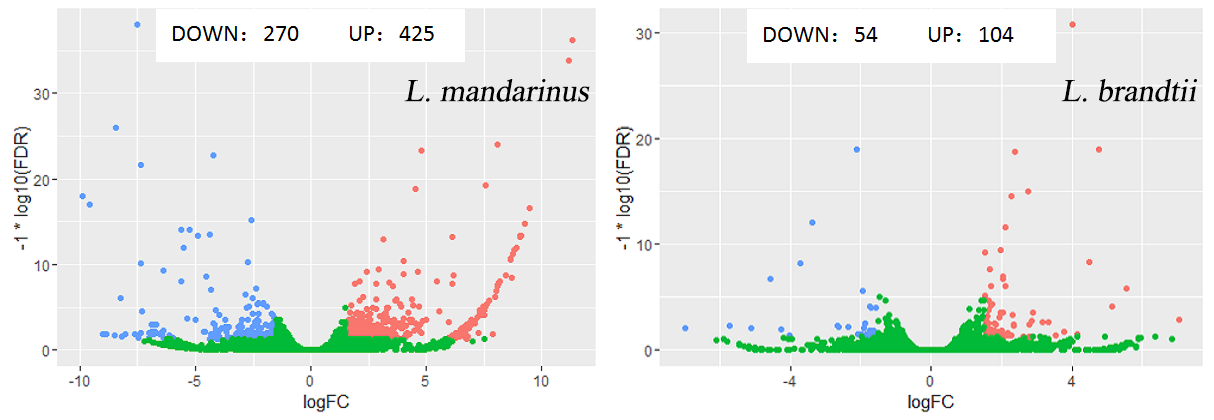

Supplement: Supplementary file 1 — Table S1. RT-qPCR primers for validation of RNA-Seq data. Table S2. Illumina sequencing data for analyzed samples. Table S3. Length distribution of assembled unigenes. Table S4. Functional annotation results for L. mandarinus and L. brandtii transcriptomes. Table S6. GO terms significant enriched for up- and downregulated DEGs in L. mandarinus and L. brandtii. Table S7. Genes associated with the GO term “peptidase activity” among up- and downregulated DEGs in L. mandarinus. Table S8. KEGG pathways enriched for up- and downregulated DEGs in L. mandarinus and L. brandtii under acute hypoxia. Figure S1. DEGs in the brain of L. mandarinus and L. brandtii under chronic hypoxia vs. normoxia. FC, fold change; FDR, false discovery rate. Red, blue, and green dots represent up- and downregulated and unchanged genes, respectively. (DOCX 481 kb) [file 12864_2018_5318_MOESM1_ESM.docx]
